# Danshen (*Salvia miltiorrhiza*) Compounds Improve the Biochemical Indices of the Patients with Coronary Heart Disease

**DOI:** 10.1155/2016/9781715

**Published:** 2016-06-05

**Authors:** Boyan Liu, Yanhui Du, Lixin Cong, Xiaoying Jia, Ge Yang

**Affiliations:** ^1^Department of Geriatrics, Affiliated Hospital, Changchun University of Traditional Chinese Medicine, Changchun 130000, China; ^2^Department of Neurology, Jilin Province People's Hospital, Changchun 130000, China

## Abstract

Danshen was able to reduce the risk of the patients with coronary heart disease (CHD), but the mechanism is still widely unknown. Biochemical indices (lipid profile, markers of renal and liver function, and homocysteine (Hcy)) are closely associated with CHD risk. We aimed to investigate whether the medicine reduces CHD risk by improving these biochemical indices. The patients received 10 Danshen pills (27 mg/pill) in Dashen group, while the control patients received placebo pills, three times daily. The duration of follow-up was three months. The serum biochemical indices were measured, including lipid profiles (LDL cholesterol (LDL-C), HDL-C, total cholesterol (TC), triglycerides (TG), apolipoprotein (Apo) A, ApoB, ApoE, and lipoprotein (a) (Lp(a))); markers of liver function (gamma-glutamyl transpeptidase (GGT), total bilirubin (TBil), indirect bilirubin (IBil), and direct bilirubin (DBil)); marker of renal function (uric acid (UA)) and Hcy. After three-month follow-up, Danshen treatment reduced the levels of TG, TC, LDL-C, Lp(a), GGT, DBil, UA, and Hcy (*P* < 0.05). In contrast, the treatment increased the levels of HDL-C, ApoA, ApoB, ApoE, TBil, and IBil (*P* < 0.05).* Conclusion*. Danshen can reduce the CHD risk by improving the biochemical indices of CHD patients.

## 1. Introduction

Coronary heart disease (CHD) is the leading cause of death in the world [1, 2]. The number of CHD patients will reach 82 million in 2020 [2]. CHD still cannot be cured and present treatment prevents symptom development and reduces the incidences of heart attacks. CHD therapy mainly includes exercise-based cardiac rehabilitation [3], the changes of the dietary patterns (stopping alcohol consumption) [4], and medication [5] as well as aortic valve replacement and coronary-artery bypass graft surgery [6]. Therefore, due to the lack of effective therapy, it is necessary to discover new treatments for preventing CHD risk.

Traditional Chinese medicine (TCM) has a profound history and has been practiced in many diseases. It is an approach to exploring new medicine and mechanism for CHD therapy [7]. Danshen (*Salvia miltiorrhiza*), a form of TCM, is often applied in the therapy for coronary heart disease [8, 9]. The results of a number of publications pointed to antioxidant [10, 11], anti-inflammatory [12], protective [13], or antiplatelet [14] properties of Danshen and its active compounds. A salvianolic acid B (SaB), an important bioactive ingredient in the root of Danshen, is being suggested to be responsible for its antioxidant property [10]. Other active water-soluble compounds, such as protocatechuic aldehyde (PAl), 3,4-dihydroxyphenyl lactic acid (DLA), and SaB with peroxides scavenging activities, were able to prevent the expression of adhesion molecules in vascular endothelium and inhibit vascular damage and the components such as tanshinone IIA and tanshinone IIB can inhibit the activity of NADPH oxidase and the aggregation of platelet [11]. This may explain the medicine usage for treating various microcirculatory disturbances. Anti-inflammatory properties of major ingredients SaB, tanshinone IIA (Tansh), and protocatechuic acid preventing the expression of adhesive molecules, cytokines, chemokines, and platelet P-selectin were also observed [12]. Furthermore, low-concentration Danshen was able to protect human umbilical vein endothelial cells (HUVECs) and improve their functions [13]. Its main components, rosmarinic acid, lithospermic acid, SaB, salvianolic acid C (SaC), D (SaD), and and H/I (SaHI), have also antiplatelet potential [14].

It is well known that the changes in the levels of a number of biochemical parameters are directly or indirectly associated with the risk of occurrence of CHD. Firstly, low-density lipoprotein cholesterol (LDL-C) is an important risk factor for CHD and the concentration should be well controlled for reducing the incidences of CHD [15], while the concentration of high-density lipoprotein cholesterol (HDL-C) is strongly and inversely associated with CHD risk [16]. A correlation with the occurrence with this disease was also observed with the changes in levels of total cholesterol (TC) and triglycerides (TG) [17, 18] as well as in the case of apolipoproteins A (ApoA), B (ApoB), E (ApoE) and lipoprotein (a) (Lp(a)) genes expression profile changes [19–22]. The markers of liver function such as *γ*-glutamyl transpeptidase (GGT) [23], total bilirubin (TBil) [24], indirect bilirubin (IBil), and direct bilirubin (DBil) [25] are also related to CHD risk. Furthermore, serum level of uric acid, one of markers of renal function [26, 27], can also reflect the severity of CHD [28]. Moreover, the high concentration of homocysteine (Hcy) concentration is regarded as a risk factor for cardiovascular disease [29, 30].

Several clinical trials showed also positive effects in the field of above-mentioned parameters, including improvement of the lipid patterns of hyperlipidemic patients [31] and protective properties in the patients with liver [32] or renal injury [33]. We hypothesized that Danshen may be able to reduce the incidences of CHD by improving these biochemical indices (lipid profile, markers of renal and liver function, and Hcy) of CHD patients. Therefore, placebo-controlled, prospective, and randomized study was conducted to investigate the effects of the medicine on biochemical indices of CHD patients and explore the possible mechanisms of its functions.

## 2. Methods

### 2.1. Patients

Before the study, all protocols were approved by the human ethical committee of Affiliated Hospital of Changchun University of Traditional Chinese Medicine. The study was conducted according to the principles of the Declaration of Helsinki [34]. All patients signed the informed consents before being enrolled in this study. From March 2011 to June 2012, 432 CHD patients attended our hospital. A total of 126 patients met following inclusion criteria and were considered for enrollment in the study.

### 2.2. Inclusion Criteria

Inclusion criteria were given according to guidelines for the management of patients with myocardial infarction [35–37]. All patients should have one of the following clinical symptoms: (1) unstable angina; (2) ST-elevation myocardial infarction (STEMI) and non-STEMI; (3) patients undergoing coronary-artery bypass grafting (CABG) surgery; (4) patients undergoing undergone percutaneous coronary intervention (PCI); patients undergoing coronary-artery stent; (5) CHD determined by angiography.

### 2.3. Exclusion Criteria

Exclusion criteria were determined according to previous reports [38–40]. The following exclusion criteria were used: (1) pregnancy and lactation; (2) renal failure with a creatinine level > 3 mg/dL; (3) multiple myeloma; (4) history of hypersensitivity; (5) cardiogenic shock or left ventricular ejection fraction < 40%; (6) patients undergoing heart transplants; (7) patients undergoing cardiac resynchronization therapy (CRT); (8) having implantable defibrillators (ICD); (9) difficult communication and other reasons.

### 2.4. Groups

Danshen compounds were extracted by ethanol and the quality was controlled according to the standard designed by China State Food and Drug Administration (http://www.sda.gov.cn/WS01/CL1236/114286.html). The main contents of ethanol extracts are tanshinone IIA, cryptotanshinone, tanshinone I [41], rosmarinic acid, and salvianolic acid B [42]. Danshen pills were the extracts of* S. miltiorrhiza* and provided as 27 mg/pill by Tianjin Tasly Group Co., Ltd (Tianjin, China). Danshen pill is composed of 0.28% tanshinone IIA, 0.21% cryptotanshinone, 0.04% tanshinone I, 1.2% rosmarinic acid, 5.8% salvianolic acid B, and most starch. After the selection of inclusion and exclusion criteria, final 126 patients were evenly and randomly assigned into two groups: Danshen group and control group. Each person was assigned to one group using an electronic spreadsheet with the indicated number. To avoid the blinding of this study, three-month run-in period was added. During the period, all patients were treated as usual. Meanwhile, to keep the stable results, the changes of lifestyle and daily food calorie intake were discouraged. CHD patients in both groups had in-person visits or telephone contact in each week. The biochemical indices were maintained constant between two groups after 3-month run-in period and then entered treatment period with Danshen.

After three-month run-in period, the patients received 10 Danshen pills/time in Dashen group [43], while the control patients received placebo pills, three times daily. Meanwhile, all patients receive the normal therapy as in run-in period and the changes of lifestyle and daily food calorie intake were discouraged. CHD patients in both groups had in-person visits or telephone contact in each week. The duration of follow-up was 3 months.

### 2.5. The Measurement of Biochemical Indices

Blood sample was obtained from the antecubital vein of each patient on the day of enrollment, after 3-month run-in period, and 3-month administration of Danshen or placebo. Serum was separated from peripheral venous blood (4 mL) after centrifuge at 4°C at 3000 rpm for 10 min. The biochemical indices were measured, including lipid profiles (LDL-C, TC, TG, HDL-C, ApoA, ApoB, ApoE, and Lp(a)), markers of liver function (GGT, TBil, IBil, and DBil), marker of renal function (UA), and a risk factor for cardiovascular disease (Hcy).

All kits were commercially available. Low-density lipoprotein cholesterol (LDL-C) BioAssay ELISA Kit (Human), Cat. number 196116, was from Beijing Huamei Scientific (Beijing, China). High-density lipoprotein cholesterol, HDL-C, ELISA Kit, Cat. number CSB-E08954h, was from Cusabio Biotech Co., Ltd (Wuhan, China). Human total cholesterol (TC) ELISA Kit, Cat. number QY-E00062, was from Qayee Bio-Technology Co., Ltd (Shanghai, China). Human TG (Triglyceride) ELISA Kit, Cat. number E-EL-H5437, was from Elabscience Biotechnology Co., Ltd (Beijing, China). Lipoprotein A (ApoA) Human ELISA Kit, Cat. Number ab108878, Apolipoprotein B (ApoB) Human ELISA kit, Cat. number ab108807, and Apolipoprotein E (ApoE) Human ELISA Kit, Cat. number ab108813, were from Abcam Trading (Shanghai) Company, Ltd (Shanghai, China). ELISA Kit for Lipoprotein (a), Lp(a), Cat. number SEA842Hu, was from Wuhan USCN Business Co., Ltd (Wuhan, China). Human gamma-glutamyl transpeptidase, GGT ELISA Kit, Cat. number E1375h, was from Everlight Biotech (Taipei, Taiwan). Total Bilirubin, Human, ELISA Kit, Cat. number E01T0143, was from ARP American Research Products, Inc (Waltham, MA, USA). Bilirubin (Total and Direct) Colorimetric Assay Kit, Cat. number K553-100, was from BioVision, Inc (Milpitas, CA, USA). Uric Acid Assay Kit, Cat. number KA1651, was from Anova Corporation (Taipei, Taiwan). Human Homocysteine (HCY) ELISA Kit, Cat. number, was from Flarebio Biotech (Wuhan, China).

Just as in a run-in period, in order to avoid the variations in biochemical indices because of normal therapy, the changes of daily food calorie intake, and lifestyle, all these changes were discouraged in three-month treatment period. After three-month follow-up, serum biochemical indices were measured on all available data. These variables still include serum lipid profiles (LDL-C, HDL-C, TC, TG, ApoA, ApoB, ApoE, and Lp(a)); serum markers of liver function, GGT, TBil, IBil, and DBil; serum marker of renal function, UA, and CHD risk factor, Hcy.

### 2.6. Statistical Analysis

A total of 126 patients (63 in each group) provided 90% power to detect the difference between two groups with an alpha level set at 0.05. All data were presented as mean values ± SD. Chi-squared test and *t*-test were applied. Analysis of variance was used to compare the serum levels of lipids at baseline and after 3-month treatment period in each group. *P* < 0.05 (2-tailed) will be regarded as statistically significant. The analysis was conducted by using SPSS version 20.0 (IBM corporation; Chicago, IL, USA).

## 3. Results

### 3.1. Baseline Characters of CHD Patients

**Figure 1:**
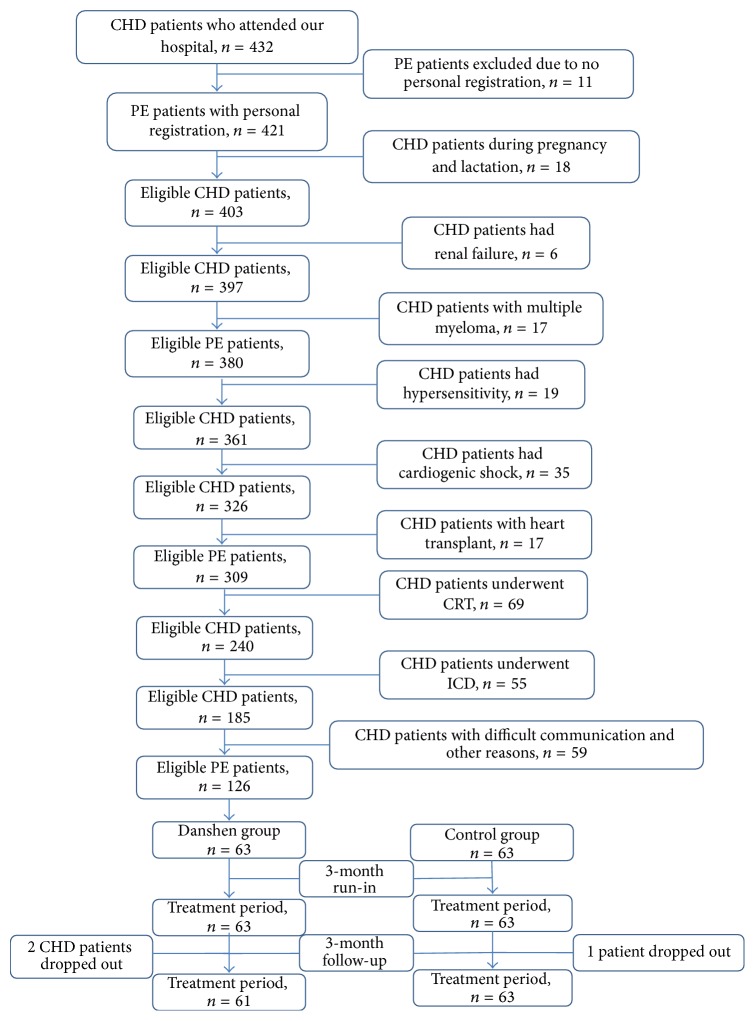
The flowchart of this study. CHD, coronary heart disease. The changes for CHD normal therapy, lifestyle, and daily food calories intake were discouraged in three-month run-in and three-month treatment periods. Finally, 61 and 62 CHD patients finished the whole procedure. Danshen pills were the extracts of* Salvia miltiorrhiza *and provided as 27 mg/pill by Tianjin Tasly Group Co., Ltd (Tianjin, China).

A total of 432 patients attended our hospital from March 2011 to June 2012. Of these patients, 306 CHD patients were excluded after selection with inclusion and exclusion criteria (Figure 1). Before administration of Danshen, 3-month run-in period was performed to make sure that there was no significant change in biochemical indices, although some of these patients are still taking the medicine for CHD therapy. Thus, 126 patients were selected and were randomly assigned to two groups: the Danshen group (*n* = 63) and the control group (*n* = 63). After another 3-month follow-up, 61 and 62 CHD patients finished the study in Danshen and control groups, respectively.

There was no significant difference for the clinical and procedural characteristics between Danshen and control groups (Table 1) (*P* > 0.05), including age, sex, risk element, clinical presentation, preprocedural laboratory results, and medication. There were 26 (41.3%) and 24 (38.1%) males in Danshen and control groups, respectively. The age of all CHD patients ranged from 60.2 to 73.5 years. Most CHD patients had unstable angina with 37 cases (58.7%) in Danshen group and 34 cases (54.0%) in control group. More than half number of patients was overweight according to BMI values (overweight = BMI of 25–29.9) in both groups [44]. Hypertension was an obvious symptom with 48 cases (76.2%) in Danshen group and 50 cases (79.4%) in control group.

### 3.2. Biochemical Indices at Baseline

Serum biochemical indices were analyzed on all available data, to primarily identify these variables associated with CHD risk. These variables include serum lipid profiles (LDL-C, HDL-C, TC, TG, ApoA, ApoB, ApoE, and Lp(a)); serum markers of liver function, GGT, TBil, IBil, and DBil; serum marker of renal function, UA and CHD risk factor, and Hcy. All serum biochemical indices between Danshen and control groups were statistically insignificant (*P* > 0.05) (Table 2).

### 3.3. Biochemical Indices after 3-Month Run-In Period

In order to avoid the variations in biochemical indices because of normal therapy, the changes of daily food calorie intake, and lifestyle, it is necessary to add three-month run-in period to make sure of the variations. Meanwhile, all these changes were discouraged. After three-month run-in period, serum biochemical indices were measured on all available data, which are associated with CHD risk. These variables still include serum lipid profiles (LDL-C, HDL-C, TC, TG, ApoA, ApoB, ApoE, and Lp(a)); serum markers of liver function, GGT, TBil, IBil, and DBil; serum marker of renal function, UA and CHD risk factor, and Hcy. The results also showed that there was no significantly statistical difference for these serum biochemical indices between Danshen and control groups after three-month run-in period (Table 3) (*P* > 0.05).

### 3.4. Analysis of Biochemical Indices after Three-Month Administration of Danshen

After three-month follow-up, two persons dropped out from Danshen group and one patient dropped out from control group. Thus, 61 and 62 patients finished the trial in Danshen and control groups (Figure 1), respectively. Danshen treatment reduced the levels of TG, TC, LDL-C, Lp(a), GGT, DBil, UA, and Hcy from median values (mg/dL) 114, 190, 113, 32, 3.3 (IU/dL), 0.4, 5.1, and 2.3 (Table 3) to media values (mg/dL) 101, 155, 98, 8, 1.6 (IU/dL), 0.2, 4.5, and 14 (Table 4), respectively (*P* < 0.05). In contrast, Danshen treatment increased the levels of HDL-C, ApoA, ApoB, ApoE, TBil, and IBil from median values (mg/dL) 55, 98, 76, 7.0, 0.5, and 0.5 (Table 3) to median values (mg/dL) 62, 119, 93, 8.7, 0.8, and 0.6 (Table 4), respectively (*P* < 0.05). Meanwhile, there were significantly statistical differences for these biochemical indices between Danshen and control groups after three-month follow-up (Table 4) (*P* < 0.05). Comparatively, there was no significantly statistical difference for these biochemical parameters in control groups between run-in and treatment periods (Tables 3 and 4) (*P* > 0.05).

## 4. Discussion

The TCM Danshen has been long regarded as effective in “activating circulation and dispersing blood stasis” [45]. According to the classic theory of TCM, it has been said that “pain will be relieved when blockage is removed.” The concept suggests that low blood circulation will do damage to human tissues and organs. Thus, such theory can be used for the management of CHD.

We assessed the therapeutic efficacy of Danshen, which is associated with the changes of lipid profiles in CHD patients. Multiple markers of biochemical indices of CHD patients were measured in the serum. The results indicated that Danshen presence may cause an improvement of several studied biochemical indices of CHD patients. Levels of TG, TC, LDL-C, Lp(a), GGT, DBil, the AU, and Hcy were statistically significantly reduced (*P* < 0.05, resp.) (Tables 3 and 4), while the levels of HDL-C, ApoA, ApoB, ApoE, TBIL, and IBil were significantly elevated (Tables 3 and 4), (*P* < 0.05, resp.). Most of these results are accordant with previous reports.

Danshen was able to improve liver function by increasing the level of total bilirubin [46] and reduce the level of UA in volunteers [47]. Hcy is a byproduct of methionine metabolism and its imbalance will result in hyperhomocysteinemia [48, 49], which is responsible for CHD development [50].* S. miltiorrhiza* extract also inhibited unwanted adverse effects for HUVECs [51]. All these results suggest that the medicine can improve heart functions and is a potential drug in CHD therapy.

Danshen has been proved to suppress the intake of low-density lipoprotein, increase the expression of intercellular adhesion molecule, and modulate key events in atherosclerosis [52]. The combination of the medicine and Gegen can improve the ratios of TG/HDL-C and LDL-C/HDL-C [53]. The main compositions in Danshen may be beneficial to the improvement of lipid profiles. Cryptotanshinone showed protective effects on atherosclerosis of ApoE-deficient mice and can improve the situation caused by apolipoprotein shortage [54], which also was able to inhibit expression of oxidized adhesion molecules induced by LDL [55]. Tanshinone IIA showed scavenging effects on lipid free radicals in cardiac sarcoplasmic reticulum [56] and inhibited expression of oxidized low-density lipoprotein receptor-1 [57]. Ethanol extract of* S. miltiorrhiza* increased in vivo serum level of HDL to prevent the occurrence of osteoporosis [58]. Lipid peroxidation prevention was also observed in the case of its active compound—rosmarinic acid [59]. All these compositions may be beneficial to the improvement of lipid profiles of CHD patients.

The incidence of CHD differs widely among different studies. The determination of degree of correlation between the risk profiles and the prevalence of factors of CHD patients is often very complicated, especially in the patients with the cooccurring diseases such as hypertension [60, 61], diabetes mellitus [62, 63], renal disease [64], and others making such results more variable. Other activities also can make CHD become worse, such as oxidative stress [65, 66] and the production of proinflammatory cytokines [67]. Thus, the prevention of these accompanying diseases and these processes may improve the clinical outcome of CHD patients. More importantly, the progression of CHD by Danshen seems to be depended on its multiple functions and beneficial effects were demonstrated in several studies. For example, the medicine has the main components with antioxidant activities [10], which can prevent vascular injury [68]. It possesses anti-inflammatory properties [12], inhibits the aggregation of platelet [69], prevents thrombosis [70], reduces blood viscosity, and improves myocardial ischemia [71]. All these results suggest that Danshen is superior to most present medicine with multiple activities, which are beneficial to improve the symptoms of CHD. However, here, we only consider Danshen improving the lipid profiles of CHD patients. Much work needs to be done to better understand its function for ameliorating the severity of CHD.

It should be emphasized that the values of obtained results even with the clinical trial's limitation could have an impact; for example, (1) the sample size seems small only with 126 selected CHD patients, which is caused by the strict criteria given in this study; (2) the whole follow-up period is 6 months, while the period for administration of Danshen is only three months. In such short period, reduction of cardiac death and heart failure cannot be detected; (3) the safety of the medicine is not identified, although it has been widely used clinically in China. Our results should be counted as a promising, although preliminary. Much more evidence is needed to support the clinical use of Danshen for CHD patients.

## 5. Conclusion

Danshen was able to improve biochemical indices of CHD patients. In a prespecified exploratory analysis, there was evidence of a reduction in the rate of CHD events among patients who had received the medicine therapy. Presently, it is the most popular Chinese herbal drug and is often used either alone or in combination with other drugs, especially for the therapies of cardiovascular diseases. Results of our study reflect the global trend of studies in the field of the role of Danshen in therapy development for CHD patients.

## Figures and Tables

**Table 1:** Baseline characters of CHD patients.

Characteristic	Danshen (*n* = 63)	Control (*n* = 63)	*P* values
Age (years)	65.9 ± 5.7	67.1 ± 6.4	0.269
Gender, male (%)	26 (41.3)	24 (38.1)	0.716
Hypertension			
Systolic blood pressure ≥ 140 mmHg or diastolic blood pressure ≥ 90 mmHg (%)	48 (76.2)	50 (79.4)	0.668
Hypercholesterolemia (>200 mg/dL) (%)	21 (33.3)	22 (34.9)	0.851
Diabetes mellitus (%)	17 (27)	14 (22.2)	0.535
BMI (kg/m^2^)	25.2 ± 5.6	25.8 ± 5.4	0.541
Cigarette smokers (%)	31 (49.2)	29 (46)	0.721
Chronic kidney disease (%)	2 (3.2)	1 (1.6)	1.000
Clinical presentation			
Unstable angina (%)	37 (58.7)	34 (54)	0.590
Non-ST-segment elevation myocardial infarction (%)	11 (17.5)	13 (20.6)	0.650
ST-segment elevation myocardial infarction (%)	12 (19)	14 (22.2)	0.660

**Table 2:** Biochemical indices measure at baseline, median (range), mg/dL.

	Danshen group (*n* = 63)	Placebo group (*n* = 63)	*P* values
Lipid profile			
LDL cholesterol	118 (95–151)	123 (94–154)	0.875
HDL cholesterol	52 (43–64)	54 (46–67)	0.436
Total cholesterol	197 (178–239)	191 (172–243)	0.527
Triglycerides	117 (85–168)	121 (84–171)	0.329
Apolipoprotein A	93 (83–126)	94 (85–128)	0.811
Apolipoprotein B	71 (62–104)	73 (65–99)	0.743
Apolipoprotein E	6.9 (5.2–8.6)	7.2 (5.3–8.5)	0.632
Lipoprotein (a)	30 (20–45)	33 (24–41)	0.237
Liver function			
Gamma-glutamyl transpeptidase (IU/dL)	2.8 (2.1–5.3)	3.0 (2.5–5.1)	0.165
Total bilirubin	0.5 (0.3–0.6)	0.6 (0.4–0.8)	0.175
Indirect bilirubin	0.4 (0.3–0.6)	0.5 (0.3–0.5)	0.268
Direct bilirubin	0.4 (0.2–0.5)	0.3 (0.2–0.4)	0.377
Renal function			
Uric acid	5.1 (4.5–6.9)	5.5 (4.1–6.4)	0.264
Risk factor of heart disease			
Homocysteine	22 (19–40)	23 (21–42)	0.459

**Table 3:** Biochemical indices measure after three-month run-in period, median (range), mg/dL.

	Danshen group (*n* = 63)	Placebo group (*n* = 63)	*P* values
Lipid profile			
LDL cholesterol	113 (90–147)	120 (91–150)	0.324
HDL cholesterol	55 (44–68)	51 (47–65)	0.512
Total cholesterol	190 (172–234)	189 (176–248)	0.763
Triglycerides	114 (87–173)	118 (82–166)	0.262
Apolipoprotein A	98 (82–126)	99 (85–128)	0.899
Apolipoprotein B	76 (66–103)	78 (62–109)	0.842
Apolipoprotein E	7.0 (5.8–8.5)	7.2 (5.4–8.8)	0.763
Lipoprotein (a)	32 (22–45)	34 (25–48)	0.268
Liver function			
Gamma-glutamyl transpeptidase (IU/dL)	3.3 (2.5–5.4)	3.1 (2.5–5.3)	0.275
Total bilirubin	0.5 (0.4–0.7)	0.6 (0.4–0.8)	0.431
Indirect bilirubin	0.5 (0.3–0.5)	0.4 (0.3–0.5)	0.176
Direct bilirubin	0.4 (0.2–0.6)	0.4 (0.2–0.5)	0.185
Renal function			
Uric acid	5.1 (4.2–6.8)	5.3 (4.2–6.4)	0.267
Risk factor of heart disease			
Homocysteine	23 (20–43)	25 (20–46)	0.341

**Table 4:** Biochemical indices after three-month follow-up, median (range), mg/dL.

	Danshen group (*n* = 61)	Placebo group (*n* = 62)	*P* values
Lipid profile			
LDL cholesterol	98 (82–133)	123 (94–157)	0.017
HDL cholesterol	62 (49–77)	50 (49–69)	0.039
Total cholesterol	155 (147–195)	192 (179–251)	0.001
Triglycerides	101 (80–158)	121 (84–172)	0.016
Apolipoprotein A	119 (103–143)	96 (81–123)	0.023
Apolipoprotein B	93 (78–114)	75 (65–101)	0.009
Apolipoprotein E	8.7 (6.8–10.7)	7.3 (5.6–8.7)	0.024
Lipoprotein (a)	18 (15–20)	31 (21–43)	0.001
Liver function			
Gamma-glutamyl transpeptidase (IU/dL)	1.6 (1.2–1.8)	3.1 (2.3–5.2)	0.001
Total bilirubin	0.8 (0.6–1.0)	0.6 (0.4–0.7)	0.032
Indirect bilirubin	0.6 (0.5–0.8)	0.4 (0.3–0.5)	0.025
Direct bilirubin	0.2 (0.1–0.3)	0.3 (0.2–0.4)	0.037
Renal function			
Uric acid	4.5 (3.6–5.5)	5.4 (4.2–6.7)	0.040
Risk factor of heart disease			
Homocysteine	14 (11–17)	24 (20–41)	0.001
